# Diagnostic utility of biomarkers in progressive supranuclear palsy: toward a biotyping framework

**DOI:** 10.1007/s00415-025-13539-6

**Published:** 2025-12-17

**Authors:** Christine Ryan, Christian Camargo, Teddy Salan, Suresh Pallikkuth, Hanzhi Gao, Varan Govind

**Affiliations:** 1 Miller School of Medicine, University of Miami, Miami, FL USA; 2 Department of Neurology, Miller School of Medicine, University of Miami, Miami, FL USA; 3 Department of Radiology, Miller School of Medicine, University of Miami, Miami, FL USA; 4 Department of Microbiology & Immunology, Miller School of Medicine, University of Miami, Miami, FL USA; 5 Health Informatics Institute, University of South Florida, Tampa, FL USA

**Keywords:** Progressive supranuclear palsy, Biotyping, Biomarkers, Fluid markers, Imaging markers

## Abstract

Progressive supranuclear palsy (PSP) is a rare and debilitating four-repeat (4R) tauopathy, characterized by motor dysfunction, cognitive decline, and oculomotor abnormalities, yet it lacks reliable biomarkers for early diagnosis, disease stratification, and prognosis. This review critically examines recent advancements in human biomarker research for PSP across multiple domains, including fluid specimen assays, i.e., blood and cerebrospinal fluid (CSF) assays, molecular profiling, and neuroimaging, with the aim of identifying markers that facilitate differential diagnosis, monitor disease progression, and support subtype classification. By synthesizing the findings of studies published, this article highlights the established biomarkers and emerging biomarkers from novel analytical methods and in vivo technologies and their potential utility for clinical trials. Emerging potential biomarkers considered include exosomal α-synuclein and tau aggregates, circulating molecular biomarkers, and relevant biomarkers from magnetic resonance spectroscopy (MRS), diffusion tensor imaging, neuromelanin MRI (NM-MRI), and functional MRI (fMRI) methods. Furthermore, the heterogeneous clinical presentations clearly reflect variable anatomical and molecular pathology in PSP and indicate that relying solely on clinical classification risks misdiagnosis, delayed treatment, and inappropriate management. A biologically grounded biotyping framework, which includes multimodal data integration and artificial intelligence, can resolve this heterogeneity by aligning observable phenotypes with underlying pathophysiology. To move beyond purely clinical classifications, this review proposes a conceptual biotyping framework to categorize PSP based on underlying biological processes, such as neurodegeneration, tau pathology, and neuroinflammation. This framework aims to guide future biomarker validation efforts, facilitate patient stratification in clinical trials, and accelerate the transition toward precision medicine approaches in PSP.

## Introduction

Progressive supranuclear palsy (PSP) is a rare progressive neurodegenerative condition that poses significant diagnostic challenges for clinicians. First described in 1964 by Steele, Richardson, and Olszewski [1], PSP affects 5–7 per 100,000 individuals, classifying it among the atypical parkinsonian syndromes [2]. Its hallmark symptoms—postural instability leading to early falls, supranuclear gaze palsy impairing vertical eye movements, and frontal–subcortical cognitive decline—set it apart from more prevalent disorders like Parkinson’s disease (PD) and Alzheimer’s disease (AD). However, these features often emerge late, and early symptoms mimic PD, corticobasal syndrome (CBS), or multiple system atrophy (MSA), resulting in diagnostic delays averaging 3–4 years and misdiagnosis rates of 30–40% [3, 4]. These diagnostic hurdles underscore the importance of the identification of reliable biomarkers to enable early detection and improve diagnostic accuracy, which will aid in the future development of disease-modifying therapies.

Pathologically, PSP is classified as a primary four-repeat (4R) tauopathy, distinguished by the accumulation of hyperphosphorylated tau proteins, forming cellular inclusions in neurons, oligodendrocytes, and astrocytes, located predominantly in subcortical structures—basal ganglia, subthalamic nucleus, substantia nigra—and brainstem, with cortical involvement in some variants [5, 6]. Yet, confirmation of these hallmarks relies on post-mortem analysis, highlighting a critical gap in diagnostics that biomarkers must bridge.

The clinical heterogeneity of PSP further complicates its management. PSP-Richardson’s syndrome (PSP-RS), the classical presentation, accounts for 50–60% of cases, marked by early falls and gaze palsy [7]. Atypical variants—PSP-parkinsonism (PSP-P), PSP-CBS, PSP-progressive nonfluent aphasia (PSP-PNFA), PSP-behavioral variant frontotemporal dementia (PSP-bvFTD), and PSP-cerebellar ataxia (PSP-C)—exhibit diverse symptom profiles, reflecting variable tau distribution [3]. Current diagnostic criteria, such as the Movement Disorder Society PSP (MDS-PSP) standards, depend heavily on clinical observation and lack a biological basis which may contribute to high misdiagnosis rates [6]. Treatments like levodopa, effective in PD, offer minimal benefit in PSP, often applied inappropriately due to diagnostic uncertainty [8, 9].

Biomarker research has surged in recent years, driven by technological advances in fluid assays, neuroimaging, and molecular profiling. Plasma and CSF biomarkers such as neurofilament light chain (NfL) and glial fibrillary acidic protein (GFAP) signal neuronal injury and glial activation, respectively. With advances in RNA sequencing and proteomics, disease-specific signatures have also been explored as an unbiased approach to screen for novel biomarkers. Neuroimaging including tau positron emission tomography (PET) with ^18^F-PI-2620 and MRI midbrain metrics also offer promising avenues for biomarker development in PSP. These advances align with the rise of precision medicine, where biomarkers stratify patients for tailored therapies, as seen in AD [10, 11]. The purpose of this review is to summarize these recent biomarker developments and propose a biotyping framework for their clinical application. With anti-tau trials (e.g., gosuranemab) underway, identifying suitable candidates and monitoring efficacy will rely on robust biomarkers [12]. PSP’s rarity and complexity demand such a system to move beyond symptomatic management toward targeted interventions.

## Definition of PSP and its clinical spectrum

PSP encompasses a spectrum of clinical presentations under the umbrella of primary four-repeat (4R) tauopathies. Understanding its pathology and diverse manifestations is essential to developing a biomarker-driven biotyping framework. PSP is a neurodegenerative disorder defined by the pathological accumulation of 4R tau isoforms [13]. Tau, a microtubule-associated protein, stabilizes axons under normal conditions, but in PSP, hyperphosphorylation detaches it from microtubules, forming insoluble aggregates [14, 15]. These manifest as neurofibrillary tangles in neurons, tufted astrocytes in gray matter, and coiled bodies in oligodendrocytes, typically concentrated in the basal ganglia, subthalamic nucleus, substantia nigra, and pontine nuclei [5, 16]. Unlike AD’s mixed 3R/4R tau or PD’s α-synuclein pathology, PSP pathology is exclusively driven by 4R tauopathy. Certain genetic factors confer risk for development of PSP, such as the H1 haplotype in the MAPT region, the gene encoding tau [17]. Ultrastructural studies confirm straight filaments, distinct from AD’s paired helical filaments, reinforcing this molecular specificity [18]. However, in vivo detection remains elusive, highlighting the need for surrogate biomarkers [19, 20]. Other primary 4R tauopathies include corticobasal degeneration (CBD), argyrophilic grain disease (AGD), and globular glial tauopathy (GGT) [21]. Clinically, CBD often presents with asymmetric limb-onset rigidity, apraxia, dystonia, myoclonus, and cortical sensory deficits (corticobasal syndrome phenotype), or occasionally Richardson’s syndrome-like features, differing from PSP’s typical midline axial rigidity, vertical supranuclear gaze palsy, and early postural instability. AGD is characterized by late-onset mild cognitive impairment with neuropsychiatric symptoms, primarily affecting medial temporal lobe structures, in contrast to PSP's prominent motor and oculomotor deficits. GGT manifests as frontotemporal dementia, pyramidal weakness (e.g., primary lateral sclerosis overlap), or a combination, with extrapyramidal features that can mimic PSP or CBD but are distinguished by prominent upper motor neuron signs [21, 22]. These overlaps in clinical presentation emphasize the diagnostic challenges and the value of biotyping to differentiate based on underlying tau pathology and distribution.

### Major clinical subtypes

PSP’s heterogeneity is well documented, with the MDS-PSP criteria delineating subtypes based on dominant features, reflecting variable tau distribution [6].

- PSP-Richardson’s syndrome (PSP-RS): accounting for 50–60% of cases, PSP-RS presents with early postural instability, falls within the first year, and supranuclear gaze palsy [7]. Brainstem and midbrain atrophy predominate, driving severe motor and oculomotor deficits [23]. Median survival is 6–7 years, reflecting rapid progression [3].
- PSP-parkinsonism (PSP-P): comprising 20–30% of cases, PSP-P mimics PD with symmetric bradykinesia, rigidity, and a transient levodopa response [7]. Tau pathology targets the substantia nigra, delaying gaze palsy and slowing progression, with survival often exceeding a decade [3].
- PSP-corticobasal syndrome (PSP-CBS): seen in 5–10% of cases, PSP-CBS features asymmetric rigidity, apraxia, and dystonia, overlapping with corticobasal degeneration (CBD) [24, 25]. Cortical tau is more prominent, complicating differentiation without biomarkers.
- PSP-progressive nonfluent aphasia (PSP-PNFA): PSP-PNFA is a rare (2–5%) subtype that manifests as effortful speech and agrammatism, linked to left frontal tau pathology [26, 27]. It mimics primary progressive aphasia variants of frontotemporal dementia (FTD).
- PSP-behavioral variant FTD (PSP-bvFTD): also, rare (2–5%), this subtype presents with disinhibition, apathy, and executive dysfunction, reflecting frontal lobe tau burden [28].
- PSP-pure akinesia with gait freezing (PSP-PAGF): PSP-PAGF is less common (4–8%), defined clinically by “freezing” during walking, writing or speaking, and difficulties with gait initiation in the absence of limb rigidity and tremor [29–31].
- PSP with predominant cerebellar ataxia (PSP-C): PSP-C is extremely rare (< 1%) with cerebellar ataxia as the primary symptom, defined pathologically by greater tau accumulation in Purkinje cells, and neuronal loss and gliosis in cerebellar dentate nucleus [ref]. PSP-C is often misdiagnosed initially as multiple systems atrophy with predominant cerebellar features (MSA-C) [32–34].

Longitudinal studies of the natural history of PSP demonstrate that only a minority of cases represent “pure” phenotypes, with most showing overlapping features at time of diagnosis [3, 35]. Among the non-RS atypical PSP phenotypes, symptoms characteristic of the PSP-RS phenotype such as supranuclear gaze palsy, postural instability, and falls, begin to emerge later in disease course [3, 35]. These findings suggest that PSP phenotypes exist along a clinical continuum and further underscores diagnostic challenges for clinicians. While early differentiation remains elusive due to clinical overlap, recent investigation of relevant biomarkers such as brainstem imaging to distinguish between PSP subtypes has been explored, yet such tools remain underutilized [20, 36]. A summary of recognized PSP phenotypes is provided in Table 1.

**Table 1:** Recognized phenotypes of PSP [37]

S. No	PSP phenotype
1	PSP with Richardson syndrome (PSP-RS)
2	PSP with predominant parkinsonism (PSP-P)
3	PSP with predominant oculomotor dysfunction (PSP-OM)
4	PSP with predominant postural instability (PSP-PI)
5	PSP with progressive gait freezing (PSP-PGF)
6	PSP with predominant frontal presentation (PSP-F)
7	PSP with predominant speech/language disorder (PSP-SL)
8	PSP with predominant corticobasal syndrome (PSP-CBS)
9	PSP with predominant cerebellar ataxia (PSP-C)
10	PSP with predominant primary lateral sclerosis (PSP-PLS)

### Misdiagnosis with Parkinson’s Disease (PD), Multiple System Atrophy (MSA), Corticobasal Syndrome (CBS), and Alzheimer’s Disease (AD)

PSP’s diagnostic challenges stem from the overlap in symptomatology with other neurodegenerative conditions, especially early in the PSP disease course. Yet, the Movement Disorder Society PSP (MDS-PSP) criteria, while refined, rely on clinical observation, yielding misdiagnosis rates of 20–40% in early stages as symptoms overlap with PD, corticobasal degeneration (CBD), and multiple system atrophy (MSA) [38]. Diagnosis is typically delayed by 2–4 years, often leading to ineffective treatments [39]. Early PSP, especially PSP-P, can mimic PD with bradykinesia, rigidity, and postural instability [2]. However, a distinguishing feature is poor response to levodopa in PSP (20–30% efficacy vs. PD’s 80–90%), and supranuclear gaze palsy, a vertical gaze limitation which is absent in PD, although this sign often emerges late and delays differentiation [38]. In addition to these parkinsonian features, dystonia represents an important diagnostic clue in PSP. Cranial dystonia including blepharospasm and cervical dystonia with retrocollis is particularly characteristic of PSP, while limb dystonia may present as the “pointing gun posture” or “gunslinger sign,” features that are less typical in PD or MSA and thus may aid differentiation [40]. PSP-CBS shares several features with CBS, another 4R tauopathy, including asymmetric rigidity and apraxia, and definitive diagnosis can only be attained through post-mortem analysis [41]. Similarly, PSP-bvFTD’s executive dysfunction and apathy resemble AD, though memory deficits are less prominent [42]. Additionally, PSP-PNFA mimics primary progressive aphasia variants of frontotemporal dementia (FTD) [26, 27]. Prominent cerebellar ataxia is a shared clinical feature of both PSP-C and MSA-C; PSP-C typically distinguished by more frequent falls and the eventual emergence of supranuclear gaze palsy, whereas MSA-C is more often characterized by early dysautonomia [32–34]. Given this lack of specificity in clinical differentiation, particularly early in the disease course, identification of imaging and fluid biomarkers represent a promising avenue to resolve these discrepancies [43, 44].

### Additional differential diagnosis and PSP mimics

Beyond classical neurodegenerative mimics, autoimmune and metabolic conditions can also resemble PSP and should be considered in the differential diagnosis. Autoimmune assays for immune-mediated neurological syndromes are particularly important, as IgLON-5 disease may present with PSP-like features, including vertical gaze palsy and gait instability [45, 46]. Unlike PSP, however, IgLON-5 disease represents a potentially treatable disorder, underscoring the clinical imperative of screening for neuronal surface antibodies in atypical presentations [45, 46]. In addition, other rare disorders can produce supranuclear gaze palsy typically associated with PSP, including Niemann–Pick type C and spinocerebellar ataxias (SCA2, SCA3, SCA35), which share oculomotor abnormalities but differ in genetic and metabolic etiology [47, 48]. Awareness of these mimics and consideration of autoimmune and genetic testing can prevent misdiagnosis and ensure appropriate management in cases that may otherwise be indistinguishable from PSP on a purely clinical basis.

## Blood-based biomarkers

Plasma biomarkers have emerged as a cornerstone in PSP biomarker research due to their accessibility, cost-effectiveness, and ability to reflect central nervous system (CNS) pathology non-invasively. Advances in ultrasensitive assays have enabled detection of low-abundance proteins, offering insights into PSP’s neurodegenerative and inflammatory processes [49]. This section evaluates key plasma biomarkers—NfL, GFAP, p-tau, amyloid-β (Aβ), and inflammatory biomarkers—for their diagnostic specificity, prognostic utility, and biotyping potential.

### Neurofilament light chain (NfL)

NfL, a cytoskeletal protein released upon axonal damage, is a robust marker of neuronal injury in PSP [50]. Plasma NfL levels are two to three times higher in PSP (60 pg/mL) than healthy control (HC) (> 30 pg/mL), reflecting subcortical and brainstem degeneration [51]. Studies show NfL correlates with PSPRS scores and survival, with higher baseline levels predicting shorter life expectancy (median 6–7 years in PSP-RS) [50, 52]. Among plasma markers including GFAP, p-tau181, amyloid-β 1–40, and amyloid-β 1–42, plasma NfL demonstrated superior performance in distinguishing PSP from healthy controls and PD, though not from MSA-P (AUC 0.624) [53]. Consistent findings by Chen et al. further highlighted NfL’s utility in differentiating PSP from PD, aiding in the differentiation of these clinically overlapping disorders [54]. When combined with MRI-based neuroanatomical metrics such as third ventricle width, and applied within machine learning frameworks, diagnostic accuracy improves further, achieving an AUC of ≥ 0.92 [55]. Given the strong discriminatory power of plasma NfL and its clinical relevance as a marker of disease severity, NfL may serve as a promising end point in therapeutic trials [53]. These studies suggest that NfL is a valuable marker for neurodegeneration and may assist in monitoring disease progression or prognosis in PSP. However, NfL lacks disease specificity, as elevated levels are also observed with normal aging and across a range of neurodegenerative disorders, including multiple sclerosis (MS), Alzheimer’s disease (AD), amyotrophic lateral sclerosis (ALS), frontotemporal dementia (FTD), multiple system atrophy (MSA), and Parkinson’s disease (PD). Moreover, substantial overlap in NfL levels between individual patients across these cohorts further limits its diagnostic utility [56–60].

### Glial fibrillary acidic protein (GFAP)

Glial fibrillary acidic protein (GFAP), an astrocytic marker, reflects glial activation in PSP. Plasma GFAP levels are approximately 1.5- to twofold higher in PSP compared to healthy controls (HC) (median: 235.5 pg/mL; range: 187.5–312.7 in PSP vs. 121.9 pg/mL; range: 91.2–156.3 in HC), and they correlate with tau deposition as measured by ^18^F-PI-2620 PET [53, 61]. However, elevated plasma GFAP levels have also been reported in other neurological disorders, including TD, CBS, DLB, and AD, highlighting its limited disease specificity [61]. This link to tufted astrocytes suggests GFAP tracks PSP’s inflammatory milieu [5]. GFAP also demonstrated the ability to distinguish PSP from multiple system atrophy-parkinsonian type (MSA-P) (AUC = 0.832) and shows a correlation with brainstem atrophy [53]. While GFAP can differentiate PSP from HC, its specificity is reduced due to overlapping glial activation in AD [61]. However, PSP’s characteristic lack of amyloid burden offers a path toward improved differentiation when GFAP is combined with Aβ biomarkers [10, 61]. As with AD, the lack of specificity in GFAP also presents a challenge when distinguishing PSP from PD, owing to shared glial activation features. Although it can distinguish tremor-dominant PD from atypical parkinsonian syndromes (APS), including PSP, it has limited utility in differentiating APS from postural instability and gait disorder dominant PD subtypes, the clinical phenotype most overlapping with PSP [54].

### Phosphorylated tau (p-tau)

Unlike AD, where p-tau181 and p-tau217 are elevated, these isoforms show minimal increase in PSP, reflecting its 4R tau profile [62, 63]. Since PSP is characterized by aggregated, insoluble tau in both neurons and glia, especially in subcortical regions, these aggregates might not lead to detectable levels of soluble pTau being released into CSF or blood. Interestingly, concentration of tau from neural-derived extracellular vesicles (NDEVs) isolated from blood provided some resolution when differentiating between PD and PSP, although this test is not widely commercially available and requires further optimization. Current assays, optimized for AD, may miss PSP-specific epitopes (e.g., Ser202) [64–66]. Together, the development of tailored assays could improve utility for p-tau, but currently supports biotyping only alongside stronger markers.

### Amyloid-β (Aβ) markers

The Aβ42/Aβ40 ratio, a cornerstone in AD, remains normal in PSP, consistent with its classification as a primary tauopathy status without amyloid plaque pathology [11]. Therefore, Aβ markers can be useful in ruling out AD in differential diagnosis, but offers little stand-alone diagnostic value for PSP [53]. However, in the context of biotyping, these markers can serve as useful adjuncts in identifying tau-predominant conditions such as PSP [62]. One study found that both Aβ42 and Aβ40 levels were decreased in atypical parkinsonian syndromes relative to PD and controls [54]. When combined with other biomarkers such as NfL and GFAP, these markers demonstrated good discriminatory power between PD and PSP (AUC = 0.884) [54].

### Inflammatory and immune markers

Blood-based testing allows assessment of immune cell populations and inflammatory changes characteristic of PSP. The neutrophil-to-lymphocyte ratio (NLR) is elevated in PSP compared to PD and controls (> 2.5 in PSP vs. 1.5–2.0 in HC), indicating a role of systemic inflammation correlating with PSPRS scores [67, 68]. In peripheral blood mononuclear cells (PBMCs) isolated from PSP patients and controls, the accumulation of phosphorylated but not total tau in patient PBMCs inversely correlated to disease severity [68]. These findings suggest that PBMC tau content could serve as a prognostic biomarker in PSP, though it has yet to be validated in vivo [68]. Tau-specific CD4 T cells have been detected in PBMC from both healthy individuals and patients with neurodegenerative diseases such as PD [69, 70]. However, tau-specific T cells have not been studied in PSP as a biomarker for disease activity. Additionally, plasma inflammatory biomarkers such as IL-6, TNFα, IL-1 β, CXCL10, MCP-1, and CRP are elevated in PSP patients, indicating ongoing systemic inflammation in PSP [71]. However, the clinical utility of these biomarkers for PSP diagnosis or disease progression is limited. Moreover, the high cost and long turnaround time of immune marker analyses limit their feasibility in routine diagnostic settings, confining their use largely to research contexts. Similarly, while the NLR has emerged as a potential biomarker of inflammatory changes in PSP, its diagnostic utility is limited by a lack of specificity. NLR is frequently elevated not only in cancers and during immune-based cancer therapies, but also in a wide range of autoimmune, neurological, and inflammatory disorders, as well as in other comorbid conditions and infections.

### Circulating molecular biomarkers

Advancements in sequencing technologies and mass spectrometry have facilitated the discovery of circulating non-coding RNAs such as microRNAs (miRNAs) and long non-coding RNAs (ncRNA) from blood and CSF in PSP. Although these RNA molecules are not translated into proteins, these markers play a critical role in regulating gene expression and cellular functions, including inflammation, neuronal integrity, and apoptosis, all of which are relevant to PSP pathogenesis. Their regulatory effects offer the potential to identify subtype-specific disease signatures and novel therapeutic targets for PSP. Distinct miRNA and ncRNA signatures found in the serum and CSF of PSP include the upregulation of hsa-let-7a-5p and hsa-piR-31068, and the downregulation of hsa-miR-92a-3p, hsa-miR-626, hsa-piR-31068, and tRNA-ValCAC [72]. Another study identified a three-miRNA panel in the serum (miR-21-3p, miR-22-3p, miR-223-5p) that distinguished PD from healthy controls, while a separate set (miR- 425-5p, miR-21-3p, miR-199a-5p) differentiated PSP from PD [73]. A systematic review also identified several key miRNAs in both serum and CSF (miR-9-3p, miR-19a, miR-19b, and miR-24) as candidate biomarkers for PSP, although further large-scale validation is required [74].

### Genomic biomarkers

Advances in molecular technologies now enable the use of biological classification frameworks rather than relying solely on clinical syndromes. In Parkinson’s disease, for example, the proposed “SynNeurGe” biological definition integrates three domains: the presence of α-synuclein pathology (S), imaging evidence of dopaminergic neurodegeneration (N), and pathogenic genetic variants (G) [75]. A comparable approach could be utilized in PSP, as genetic studies have revealed several susceptibility variants that could be used to build a biological taxonomy for PSP diagnosis. Although PSP is predominantly considered a sporadic condition, rare cases with familial inheritance have been described. Among genetic influences, the common MAPT H1 haplotype is recognized as the strongest genetic risk factor for PSP [76]. Beyond MAPT, genome-wide association studies (GWAS) have highlighted additional loci of interest within the MOBP gene (myelin-associated oligodendrocyte basic protein) has been linked to PSP, though this association was shared with CBD [77]. Another GWAS study identified variants associated with PSP including EIF2AK3 and STX6 (syntaxin 6) related to endoplasmic reticulum (ER) stress and endosomal and vesicular recycling which are known to be dysregulated in PSP [78]. More recently, additional risk loci were identified in SLCO1A2, an organic anion transporter expressed at the blood–brain barrier, and DUSP10, a phosphatase regulating MAPK signaling pathways, in PSP. These findings expand the genetic landscape of PSP beyond tau-related mechanisms, implicating broader cellular pathways in disease susceptibility [79]. Incorporating genomic information into diagnostic frameworks for PSP represents a promising strategy, particularly when used in a combinatorial approach like the “SynNeurGe” model in PD, though current data lacks sufficient resolution in differentiating PSP from other neurodegenerative diseases such as PD, and among different PSP phenotypes, highlighting an important area for future investigation.

## Cerebrospinal fluid (CSF) biomarkers

CSF offers a direct window into central nervous system (CNS) pathology, making it a critical medium for identifying biomarkers. Unlike plasma, CSF’s proximity to brain tissue captures disease-specific changes with greater sensitivity, though its invasive collection limits routine use [80]. Advances like ultrasensitive assays and harmonized cutoffs could bridge this gap, positioning CSF as a gold standard for validating plasma findings and informing PSP biotypes [49].

### CSF neurofilament light chain (NfL)

CSF NfL, a marker of axonal damage, is markedly elevated in PSP (median > 1,400 pg/mL vs. < 500 pg/mL in HC), secondary to subcortical and brainstem degeneration [81, 82]. Longitudinal studies show that the increase in CSF NfL parallels motor decline [81, 83]. However, the ability of NfL as a stand-alone biomarker to distinguish between PSP and FTD is still controversial. One systematic review showed conflicting evidence with most studies showing higher or similar levels of CSF NfL in FRTD relative to PSP, although one study also showed lower levels compared to PSP [84, 85]. NfL differentiates PSP from PD and MSA with AUC values of 0.85–0.90, though overlap with CBD (also a 4R tauopathy) requires additional markers [86, 87].

### Total tau (t-tau) and p-tau

Unlike AD, which is characterized by elevated CSF p-tau, PSP shows an inverse correlation between CSF p-tau levels and both disease severity and progression rate, suggesting a mechanistically distinct tau pathology from that observed in AD [81]. Predictive accuracy for disease severity was further enhanced by the CSF NfL/p-tau ratio, which outperformed either biomarker alone [81]. Another assay targeting N-terminal epitopes of total tau, no differences were detected between PSP and control subjects [88]. These results are consistent with a meta-analysis which found that PSP subjects had decreased levels of p-tau in CSF with no significant differences in t-tau [89]. However, paired with NfL or tau PET, CSF measurements of t-tau/p-tau could refine tau-predominant biotypes.

### Neuroimmune markers

CSF analysis of inflammatory markers can offer insight into the neuroimmune dynamics of PSP and identify novel biomarkers. IL-13 was identified in the analysis of post-mortem tissue able to distinguish between PSP and AD or CBD independent of age or gender, but was not assessed in CSF to assess eligibility as future biomarker [90]. Proteomic analysis of CSF in PSP revealed two immune cell-derived inflammatory proteins that correlated with disease severity as measured PSPRS: galectin-10, which showed a positive correlation, and cytotoxic T-lymphocyte-associated protein 4 (CTLA-4), which exhibited a negative correlation [91]. However, this comparison was between PSP and controls only which limits the resolution of this analysis to identify between PD, and similar diseases [91].

### Exosomal α-synuclein and tau aggregates

Exosomes are CNS-derived vesicles that concentrate pathological proteins, allowing for detection. CSF exosomal tau aggregates are elevated in PSP, with seeding assays distinguishing it from PD (AUC ~ 0.85) and reflecting subcortical tauopathy [92]. Exosomal α-synuclein, lower in PSP than PD/MSA, aids differential diagnosis, though levels overlap with CBD [92, 93]. These markers, while promising, require standardized isolation protocols to overcome variability. In biotyping, these markers could define tau- versus synuclein-driven PSP variants.

### Circulating molecular biomarkers

Advancements in sequencing technologies and mass spectrometry have facilitated the discovery of circulating non-coding RNAs such as microRNAs (miRNAs) and long non-coding RNAs (ncRNA) in PSP. Although these RNA molecules are not translated into proteins, these markers play a critical role in regulating gene expression and cellular functions, including inflammation, neuronal integrity, and apoptosis, all of which are relevant to PSP pathogenesis. Their regulatory effects offer the potential to identify subtype-specific disease signatures and novel therapeutic targets for PSP. Distinct miRNA and ncRNA signatures found in the serum and CSF of PSP include the upregulation of hsa-let-7a-5p and hsa-piR-31068, and the downregulation of hsa-miR-92a-3p, hsa-miR-626, hsa-piR-31068, and tRNA-ValCAC [72]. Another study identified a three-miRNA panel (miR-21-3p, miR-22-3p, miR-223-5p) that distinguished PD from healthy controls, while a separate set (miR-425-5p, miR-21-3p, miR-199a-5p) differentiated PSP from PD [73]. A systematic review also identified several key miRNAs (miR-9-3p, miR-19a, miR-19b, and miR-24) as candidate biomarkers for PSP, although further large-scale validation is required [74].

## Neuroimaging biomarkers

Neuroimaging provides a non-invasive lens into PSP’s structural, molecular, and metabolic alterations, leveraging advances in magnetic resonance imaging (MRI), positron emission tomography (PET), and magnetic resonance spectroscopy (MRS). These tools visualize PSP’s hallmark midbrain atrophy, tau deposition, and neuroinflammation, distinguishing it from HC, PD, MSA, CBD, and AD with high accuracy. Their quantitative nature supports early diagnosis, progression tracking, and biotype delineation.

### Structural MRI

**Fig. 1:**
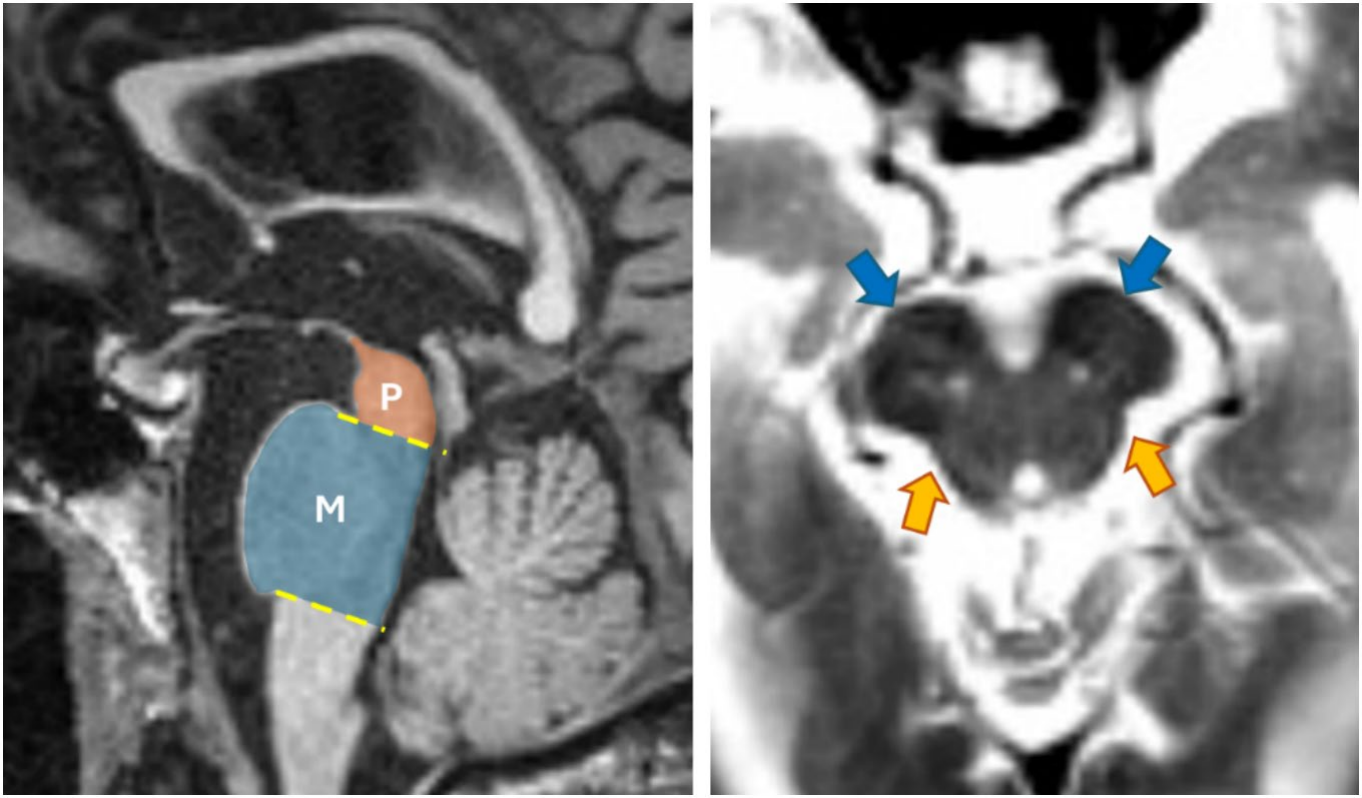
On the left, a T1-weighted midsagittal MR image showing the hummingbird sign with atrophy of the dorsal midbrain, with segmentation of the midbrain (M) and pons (P) in a patient with progressive supranuclear palsy. On the right, a T2-weighted axial MR image showing the Mickey Mouse sign (blue arrows—atrophy of the midbrain tegmentum resembling the head of a Mickey Mouse) and morning glory sign (red arrows—concavity of the lateral margin of the midbrain tegmentum) in the same patient

Midbrain atrophy is a hallmark imaging feature in PSP. It can be visually identified and often referred to as the “hummingbird”, “Mickey Mouse”, and “Morning Glory” (Fig. 1), and can also be quantified by the midbrain-to-pons (M/P) area ratio, the Magnetic Resonance Parkinsonism Index (MRPI), in addition to other clinically useful measurements. An M/P ratio of < 0.52 (vs. > 0.60 in healthy controls and PD) yields ~ 90% sensitivity and specificity and can precede the onset of vertical gaze palsy by several years [94, 95]. The MRPI further enhances diagnostic precision by incorporating M/P along with the ratio of middle to superior cerebellar peduncle widths [(P/M) × (MCP/SCP)] [96, 97]. MRPI values are significantly elevated in PSP (median > 19) compared to MSA-P (median 6.53) and controls (median 9.21), demonstrating excellent diagnostic accuracy (sensitivity and specificity 100%) and the ability to predict the emergence of vertical supranuclear gaze palsy at an individual level [96, 97].

The M/P ratio and MRPI not only distinguish PSP from other parkinsonian syndromes, but also reveal variation across PSP subtypes [36]. The most pronounced abnormalities in these brainstem metrics are seen in PSP-Richardson’s syndrome and frontal variant PSP, followed by PSP-corticobasal syndrome (PSP-CBS), PSP with predominant speech/language disorder (PSP-SL), and PSP-parkinsonism (PSP-P) [36]. In contrast, the PSP with predominant gait freezing (PSP-PGF) variant tends to show normal M/P and MRPI values, reflecting minimal brainstem atrophy on imaging [36]. These subtype-specific differences underscore the utility of quantitative MRI in both diagnosis and phenotypic characterization of PSP.

MRPI 2.0 is an enhanced imaging metric that integrates third ventricle width into the original MRPI formula by multiplying MRPI by the ratio of third ventricle width to frontal horn width [98]. This revised index has demonstrated particular utility in distinguishing PSP-parkinsonism (PSP-P) from PD, especially in early stages without vertical supranuclear gaze palsy (VSGP), but with slowed vertical saccades [98]. While both MRPI and MRPI 2.0 showed high specificity (> 94%) in differentiating PSP-P from PD, MRPI 2.0 achieved superior sensitivity (100% vs. 73.5%), making it a valuable diagnostic tool in challenging cases [98].

Newer studies utilizing artificial intelligence (AI)-based MRI analysis tools differentiate PSP from both controls and PD and identify specific brain regions and fiber tracts that could support clinically meaningful classification accuracy [99]. Among the models, the random forest machine learning (ML) algorithm and deep learning (DL) neural network achieving classification accuracies of > 90% in PSP from control with key brain regions including pons, midbrain tegmentum, superior cerebellar peduncle, putamen, and corpus callosum [99]. The DL model achieved > 85% accuracy when comparing PSP to PD with the most informative features being DTI metrics in the prefrontal white matter, fronto-occipital fasciculus, midbrain tegmentum, and corpus callosum [99].

### PET

Positron emission tomography (PET) imaging can be used to visualize brain activities and processes at molecular and cellular levels using specific radiotracers. Radiotracers commonly used include ^18^F-fluorodeoxyglucose (^18^F-FDG) for measuring glucose metabolism, and ^18^F-flortaucipir ^18^F-PI-2620 and APN-1607 for visualizing tau deposits and aggregates in the brain of patients with suspected PSP. FDG-PET frequently shows hypometabolism in several brain regions in PSP that include the frontal cortex, insular cortex, caudate nucleus, putamen, brainstem, and cerebellum [100, 101]. FDG-PET can help distinguish PSP from other parkinsonian syndromes (e.g., Parkinson’s disease and multiple system atrophy) and aid in earlier diagnosis in some cases with inconclusive or normal MRI findings; however, it lacks standardized quantitation methods [20] and yields inconclusive findings in approximately one-third of cases [102], necessitating the use of an additional PET scan with tau-specific PET tracers. First generation of tau PET tracers for PSP [103] including ^18^F-flortaucipir (aka AV1451 or T807), ^18^F-THK5351, ^18^F-THK5317, and ^11^C-PBB3 have shown to selectively target and visualize 4R tau deposits due to off-target binding and inconsistency [103].

Second-generation tracers like ^18^F-PI-2620 target PSP’s 4R tau with high affinity. A study by Brendel et al. [104] demonstrated that ^18^F-PI-2620 PET imaging showed significantly higher binding in PSP target regions, including the globus pallidus internus (GPi), subthalamic nucleus, and substantia nigra, compared to controls, and reported mean distribution volume ratios (DVRs) of 1.21 in GPi in PSP (vs. 1.00 in controls). Unlike ^18^F-flortaucipir, which exhibits off-target binding and does not correlate with long-term PSPRS scores, ^18^F-PI-2620 distinguishes PSP from AD (cortical tau) and PD (no tau) with AUC ~ 0.90 [105]. Its specificity for tau-predominant biotypes may enhance the design of clinical trials, though cost and availability limit routine use. Oliveira Hauer et al. have explored the utility of tau PET tracer ^1^⁸F-RO948 uptake in the GPi alongside structural MRI and CSF NfL levels and found that combining these three biomarkers enhanced accuracy (AUC > 0.90) in distinguishing PSP from both controls and alpha-synucleinopathies (PD, Lewy body dementia) [106]. Another study [107] evaluated a novel PET tracer, ^18^F-THK-5351, and found a higher tracer retention in globus pallidus and midbrain in patients with PSP.

Tracers like ^11^C-PK11195 bind translocator protein (TSPO) on activated microglia, revealing elevated regional uptake in PSP’s basal ganglia and brainstem (vs. controls and AD) [108]. ^11^C-PK11195 tracer signals show co-localization with regions of tau pathology, and when analyzed in combination, these markers can predict clinical progression and disease severity [109, 110].

### Dopamine transporter (DAT) imaging

Novel PET is capable of binding presynaptic dopamine transporters and thereby serve to visualize alterations to dopaminergic transmission and function in PSP and related diseases. One such study utilizing ^11^C-CFT PET compared spatial patterns of dopaminergic dysfunction among HC, PD, MSA-P, and PSP (Qi-Si Chen et al.). PSP exhibited greater DAT degeneration in the striatum compared to PD and MSA-P, particularly in the caudate, though DAT loss was more severe in the putamen than caudate across all groups. Notably, DAT imaging was unable to differentiate among PSP subtypes. A separate study using ^1^⁸F-FP-CIT PET similarly identified regional patterns of DAT reduction, showing earlier and more severe DAT loss in the anterior caudate in PSP, in contrast to PD, where DAT loss was predominantly observed in the posterior caudate. Additionally, MSA-P cases showed more prominent DAT reduction in the ventral putamen compared to PSP, further supporting region-specific differences across parkinsonian syndromes. Investigations into the laterality of striatal DAT binding using ^123^I-FP-CIT SPECT showed PSP patients typically exhibit less lateralized DAT loss compared to early-stage PD patients (Hoehn-Yahr stage 1) but not later stages, suggesting asymmetry measurements may have less discriminatory power in later stages of PD relative to PSP [111]. More recently, the DAT PET tracer ^18^F-LBT-999 has been extensively used in rodent and non-human primate models demonstrating its capability to assess dopaminergic presynaptic injury and neuron loss in physiological conditions such as lesion-induced rat models of PD and to evaluate the beneficial effects of therapeutic approaches such as pharmacological treatment and cell transplantation [112]. Clinical trials are underway evaluating the sensitivity of ^18^F-LBT-999 for distinguishing PD from other disorders, compared to existing DAT methods [113].

### Whole-brain single photon emission computed tomography (SPECT)

Whole-brain SPECT imaging serves as a valuable adjunct in evaluating atypical parkinsonian syndromes (APS), particularly in differentiating them from idiopathic PD. By utilizing gamma-emitting radionuclides such as ^123^I and ^93m^Tc, this modality enables the assessment of regional cerebral perfusion, which serves as a surrogate marker of neuronal function and metabolism [114, 115]. Striatal hypoperfusion is commonly observed in most atypical forms of parkinsonism and can help distinguish these disorders from idiopathic PD [116]. However, PSP perfusion deficits are typically seen in frontal lobes which have been linked to impaired cognitive performance in PSP patients [117, 118]. While perfusion SPECT can reveal functional abnormalities in PSP, its specificity is limited due to overlapping perfusion patterns with other neurodegenerative disorders. Therefore, perfusion SPECT findings should be interpreted in conjunction with clinical assessments and other imaging modalities.

DAT and SPECT imaging are often used to exclude PD and other movement disorders mimicking PSP symptoms and signs, but these imaging methods lack specificity to PSP and therefore are not always considered conventional imaging for PSP. The findings from these modalities are used to determine if there is a dopaminergic deficit to provide supportive evidence towards neurodegenerative parkinsonism.

## Emerging neuroimaging biomarkers

Several imaging modalities were not explicitly noticed in our review of the literature. However, we have included select studies for consideration here, as these emerging modalities may provide future biomarker candidates in PSP.

### Magnetic resonance spectroscopy (MRS)

MRS utilizes proton magnetic resonance spectroscopy (^1^H-MRS) to detect metabolic shifts and characterize biochemical changes in various regions across disease states. One study focused on biochemical changes in the supplementary motor area (SMA), finding a significant reduction in scyllo-inositol levels (s-Ins) and s-Ins/Cr ratios in PSP patients relative to controls [119]. Additionally, in PSP patients, higher s-Ins and s-Ins/Cr values were associated with better cognitive performance [119]. The authors speculate that because s-Ins may inhibit amyloid-beta and tau aggregation, these results suggest its potential as a therapeutic target as well as biomarker in PSP [119, 120].

### Diffusion tensor imaging along the perivascular space (DTI-ALPS)

The glymphatic system acts as the brain’s waste clearance system and is responsible for the removal of toxic waste products and molecules from the central nervous system [121, 122]. Glymphatic impairment has been observed in several subcortical diseases such as PSP, AD, PD, and CBS [123–126], and have been shown to associate with cortical tau deposition tau and cognitive dysfunction [127]. In recent years, diffusion tensor imaging (DTI) along the perivascular space (DTI‐ALPS) has emerged as a non‐invasive neuroimaging technique capable of assessing glymphatic function by quantifying water diffusivity along perivascular spaces (better known as the DTI-ALPS index) [128, 129]. The DTI‐ALPS index is a diffusion imaging‐derived metric designed to assess glymphatic function. Studies have consistently shown lower DTI-ALPS index in PSP patients compared to matched controls, as well as potential in differentiating PSP from other neurodegenerative disorders [130].

### Advanced diffusion imaging methods

Conventional DTI is widely used in clinical practice and research for assessing white matter microstructure; however, it has significant limitations in modeling complex fiber architectures, non-Gaussian diffusion, and diffusion within multiple tissue compartments. More advanced methods have been developed to address these challenges: free-water imaging uses a two compartment to model the intra-cellular water and extra-cellular water (or free-water) diffusion separately and obtain an estimate of the free-water volume fraction [131]; Neurite orientation dispersion and density imaging (NODDI) [132, 133] uses a three-compartment model for glial cells, axons, and extra-cellular space and incorporates exchange of water between the two intra-cellular and the extra-cellular compartments; Fixel-based analysis (FBA) uses fiber density and orientation to better quantify white matter properties and changes [134]. A multi-site longitudinal study using these three methods showed high sensitivity to widespread and differential white matter changes in PSP, MSA, and PD [135], associated with changes in clinical disease severity across all three parkinsonian syndromes over a 1-year period. FBA and free-water imaging revealed longitudinal declines in a greater number of descending sensorimotor tracts in MSA and PSP compared to PD. PSP was also characterized by longitudinal impairment in multiple transcallosal tracts measured by FD, whereas no such impairments were seen in MSA and PD. Other studies using FBA also identified distinct patterns of white matter degeneration between patients with atypical parkinsonism (MSA and PSP) compared to PD [136], and disease-specific progression patterns of fiber density loss and subsequent bundle atrophy between PSP and CBS [137].

### Neuromelanin-MRI (NM-MRI)

Neuromelanin-sensitive MRI (NM-MRI) purports to detect the content of neuromelanin (NM), a product of dopamine metabolism that accumulates with age in dopamine neurons of the substantia nigra [138]. NM-MRI has since been used as a proxy measure of dopaminergic neuronal degeneration underlying neurodegenerative diseases including PSP [139]. A reduction in the neuromelanin volume and signal-to-noise ratio of the substantia nigra pars compacta was found in PSP [140]. Findings have also shown strong correlation between NM-MRI and midbrain volumetry as clinical and pathological characteristics of PSP. Combining neuromelanin-sensitive MRI and midbrain volumetry can therefore be useful for differentiating PSP from other diseases.

### Functional MRI (fMRI)

fMRI captures changes in cerebral blood flow and oxygenation, which can be used to infer patterns of functional connectivity. Resting-state fMRI assesses spontaneous co-activation across brain regions, revealing intrinsic functional networks. In PSP, significant disruptions to the cerebello-thalamo-cortical network have been reported and can be utilized to distinguish it from similar diseases [141, 142] For example, while thalamic connectivity is similarly reduced in both PSP and CBD relative to controls, dentate connectivity decreases in PSP and increases asymmetrically in CBS [141]. Additionally, alterations in midbrain network connectivity have been observed and correlated with impairments in both cognitive and motor function [143, 144]. All the neuroimaging techniques used and their primary features in PSP are summarized in Table 2.

**Table 2:** Summary of neuroimaging modalities used and PSP-specific biomarkers

Imaging type	Primary features in PSP
Structural MRI	Hummingbird, Mickey Mouse, and morning glory signs
	Higher MRPI and lower M/P ratio
	M/P ratio < 0.52 strongly suggestive of PSP
	Automated volumetric analysis may improve specificity
PET	Glucose hypometabolism (^18^F-FDG) and tau deposits (^18^F-PI-2620 and APN-1607) in several brain regions including frontal cortex, insular cortex, caudate nucleus, putamen, brainstem, and cerebellum
DAT	DAT reduction in the striatum, putamen, and anterior caudate
SPECT	Striatal and frontal lobe hypoperfusion
MRS	Significant reduction in scyllo-inositol (s-Ins) and s-Ins/Cr ratio
DTI-ALPS	Lower DTI-ALPS index in PSP compared to PD and controls
NM-MRI	Lower NM signal-to-noise ratio
fMRI	Alterations in midbrain network connectivity

## The promise of biotyping in PSP

**Fig. 2:**
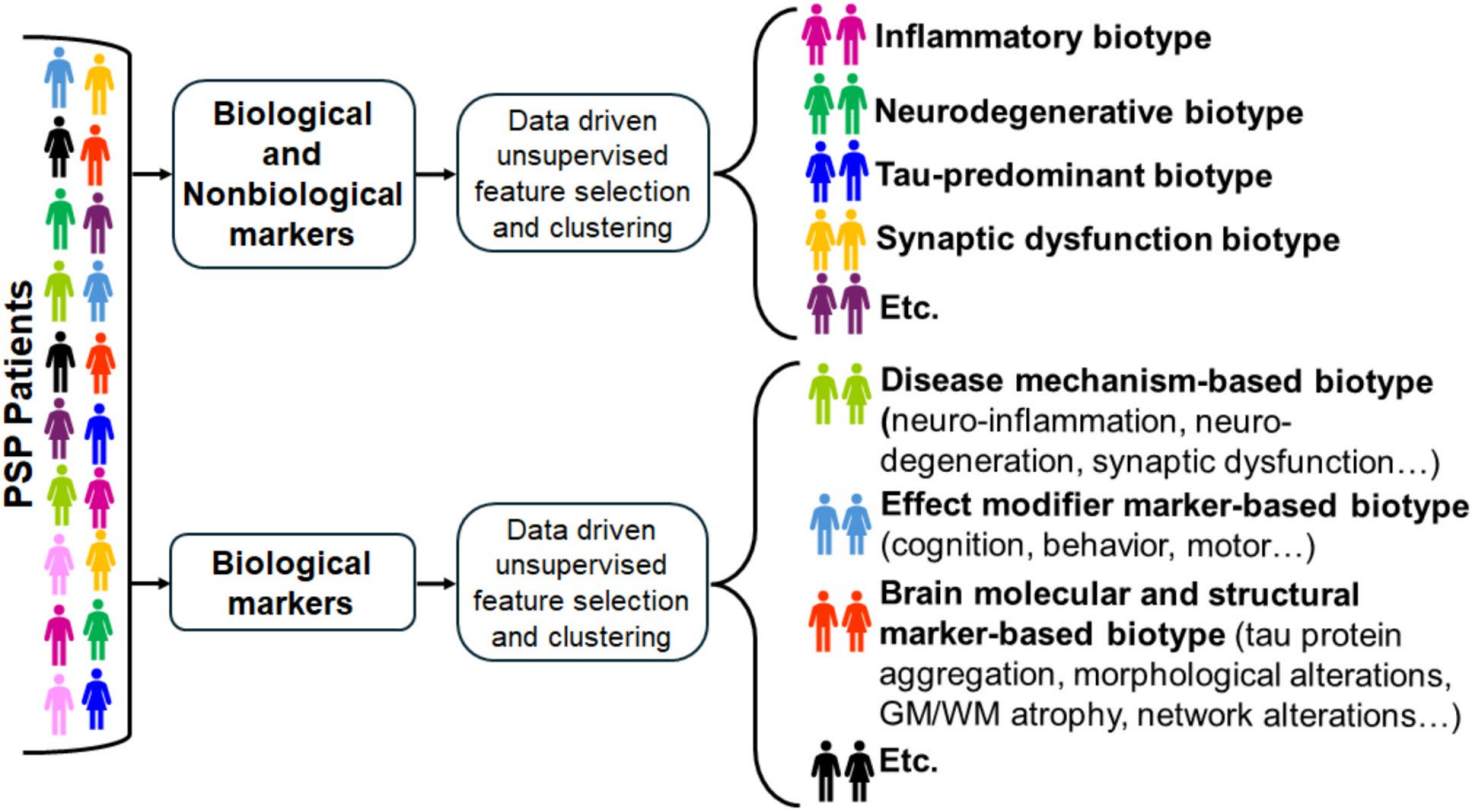
Biotyping framework

The heterogeneous clinical presentations of PSP reflect variable anatomical and molecular pathology [3]. While clinical classification remains essential, relying solely on it may contribute to diagnostic challenges and delays in accurate diagnosis. This reliance can lead to management that could be more precisely tailored, such as through prolonged levodopa trials at high dosages of at least 900–1000 mg daily for several months, which may provide limited or transient benefits in a subset of patients and are often assessed via washout periods. A biologically grounded biotyping framework can resolve this heterogeneity by aligning observable phenotypes with underlying pathophysiology (Fig. 2).

The biotyping model offers multiple applications in diagnosis, disease monitoring, and treatment of PSP. For example, MRI-based midbrain-to-pons (M/P) ratios below 0.52 and elevated levels of plasma or CSF neurofilament light chain (NfL) can help distinguish PSP from PD and multiple system atrophy (MSA), even in early or clinically ambiguous cases [36]. Biotyping may also be valuable for monitoring disease progression. Longitudinal changes in biomarker levels offer objective metrics that can complement clinical tools like the PSP Rating Scale (PSPRS), which may lack sensitivity to subtle or early-stage changes [105, 145]. Finally, biomarkers can guide therapeutic development by enabling stratification by molecular markers. Identifying patients with high tau burden or prominent glial activation allows for targeted enrollment in clinical trials, reducing heterogeneity and increasing the likelihood of detecting treatment effects. This biotyping approach could renew interest in therapies such as gosuranemab, which failed in unstratified PSP cohorts [12] (Fig. 2).

### Potential PSP Biotypes

There are various approaches described in the literature [146–152] for classifying both the above established and emerging biomarker profiles [140], which can be used to develop a taxonomy of distinct PSP biotypes. For example, biotypes may be defined based on combinations of mechanistic biomarkers, pathological biomarkers, and effect modifiers. Alternatively, some classification systems may use only pathological biomarkers, such as those related to inflammation, neurodegeneration, and alterations in cerebral cellular microstructure. Each biotyping framework offers unique ramifications for both clinical diagnosis and therapeutic decision-making.

- Inflammatory biotype: characterized by high NLR (> 2.5), and ^11^C-PK11195 PET uptake, this biotype reflects robust microglial activation and systemic inflammation [67, 108, 109]. The inflammatory biotype may respond to trials of immunomodulatory drugs (e.g., minocycline) [153–155].
- Neurodegenerative biotype: marked by high plasma/CSF NfL (> 30 pg/mL, > 2000 pg/mL) and low M/P ratio (< 0.52), this biotype signals extensive axonal loss and midbrain atrophy [36, 51]. This proposed biotype could be amenable to treatment with neuroprotective agents and longitudinal NfL monitoring to track efficacy.
- Tau-predominant biotype: defined by high ^18^F-PI-2620 uptake (SUVR > 1.5) and normal Aβ42/Aβ40, this biotype highlights PSP’s 4R tauopathy without amyloid co-pathology [20, 105]. Anti-tau therapies would represent an ideal candidate, with tau PET used to monitor treatment response [156].
- Synaptic dysfunction biotype: low scyllo-inositol on MRS and altered CNTN1 in proteomics indicate synaptic loss, potentially prominent in PSP-PNFA or PSP-bvFTD [119, 157]. This biotype could guide synaptic repair strategies, though overlap with AD requires refinement.

## Discussion

Biomarker research in PSP has rapidly evolved, identifying diverse molecular, imaging, and fluid-based candidates that promise to reshape the clinical landscape of this challenging 4-repeat tauopathy. However, translating these discoveries into diagnostic and therapeutic frameworks remains constrained by methodological inconsistencies, disease biomarker overlap, and the lack of large, longitudinal validation studies. This discussion synthesizes these barriers, rationalizes the need for a biomarker-based biotyping approach, proposes biologically grounded PSP subtypes, and outlines key steps toward aligning PSP management with precision medicine.

Lack of standardization remains a critical obstacle in PSP biomarker research. Plasma NfL, one of the most widely studied biomarkers in PSP, is measured using different platforms (i.e., ELISA, Simoa) that yield substantially different concentration thresholds. For example, Simoa-based high sensitivity detection typically reports lower values than traditional ELISA, complicating cross-study comparisons and limiting clinical translation [158]. However, high sensitivity assays may facilitate a more reliable interpretation of results, as these can detect low concentrations in healthy donors, in contrast to those observed in disease states. Consequently, the levels detected in disease states can be reported with greater confidence, even when these markers are only marginally elevated above the normal range. Tau PET ligands present a similar issue: while ^1⁸^F-AV-1451 shows extensive off-target binding in PD and multiple system atrophy (MSA), newer tracers like ^1⁸^F-PI-2620 offer improved specificity for 4R tau, yet their clinical use is limited by issues with standardization and accessibility [159]. Additionally, technical protocols for CSF collection and processing, miRNA isolation, and biomarker detection platforms vary widely across laboratories, further impairing reproducibility and clinical application [160, 161]. Beyond technical variations, biomarkers of PSP can also overlap with other neurodegenerative diseases that reduce diagnostic precision. Elevated NfL levels are also found in CBD, FTD, and MSA, reducing its utility as a standalone discriminatory marker for PSP [82]. Tau PET imaging can distinguish PSP’s subcortical tau deposition from the cortical pathology typical of AD, but this distinction diminishes when compared with other 4R tauopathies like CBD, which share similar anatomical distributions and binding profiles [162]. Likewise, inflammatory PET tracers such as ^11^C-PK-11195 highlight microglial activation across PSP, CBD, and MSA, limiting disease-specific interpretation [108, 163–166]. These limitations underscore the inadequacy of relying on single biomarkers in isolation. Instead, these limitations highlight the need for integrated, multimodal approaches that combine fluid-based markers (e.g., NfL, GFAP), structural MRI indices (e.g., MRPI), and molecular imaging (e.g., tau PET) to enhance diagnostic specificity [55, 105, 167]. Studies applying machine learning frameworks to such multimodal data have also shown encouraging accuracy rates, reinforcing this integrative strategy [99, 168].

Recent advances in biotyping methods have employed diverse data-driven approaches to identify homogeneous subgroups across traditional diagnostic boundaries. The framework involves unsupervised machine learning pipelines consisting of feature selection via variance thresholding and false discovery rate correction, dimensional reduction through principal component analysis to extract independent biomarkers, and unsupervised clustering methods, ranging from hierarchical clustering [169] to K-means non-hierarchical clustering [152, 170–174] followed by validations using independent datasets. These clustering approaches have been applied to various biological modalities, including structural MRI-derived data, cognitive data, as well as inflammatory proteome data. To enhance clinical translation, supervised machine learning methods have also been implemented for biotyping, such as growth mixture modeling [175], latent profile analyses [176], and regression models [177]. These methods collectively demonstrate a shift from exploratory clustering to clinically implementable algorithms, with increasing emphasis on minimizing assessment burden while maintaining diagnostic accuracy. Emerging evidence suggests that some biomarkers may also carry prognostic value multiple studies have shown plasma and CSF NfL levels correlate with disease severity and survival. However, the lack of long-term studies impairs prognostic value [50, 51, 81]. Longitudinal studies tracking dynamic biomarker changes are essential to understanding progression patterns, particularly across the clinical subtypes of PSP. Current longitudinal efforts, such as those in AD under the AT(N) framework, serve as blueprints for biomarker-guided staging but remain underdeveloped in PSP [10].

Finally, a significant limitation of the studies cited in this review is that many lack pathological confirmation, relying instead on clinical diagnostic criteria such as the MDS-PSP standards. Post-mortem examination remains the gold standard for definitive diagnosis of PSP, as it confirms the presence of 4R tau pathology and rules out overlapping conditions like corticobasal degeneration or other tauopathies. While some studies incorporate autopsy-confirmed cohorts to validate biomarkers (e.g., in neuroimaging or fluid marker correlations), the absence of this in the majority of prospective and cross-sectional research may introduce diagnostic uncertainty and affect the generalizability of findings. Future biomarker validation efforts should prioritize pathologically confirmed cases to strengthen the evidence base and support the transition to precision medicine in PSP [6, 178, 179].

## Conclusion

A biomarker-driven biotyping system holds transformative potential for PSP. It could address the disorder’s heterogeneity, improve diagnostic precision, prognostic value and provide a roadmap for tailored therapeutic strategies. Large scale implementation of biotyping demands continued investment in biomarker standardization, multimodal integration, and longitudinal validation. By mapping biology to phenotype, biotyping aligns PSP with precision medicine, enhancing diagnosis, prognosis, and treatment.
